# Rpb5, a subunit shared by eukaryotic RNA polymerases, cooperates with prefoldin-like Bud27/URI

**DOI:** 10.3934/genet.2018.1.74

**Published:** 2018-02-27

**Authors:** Verónica Martínez-Fernández, Francisco Navarro

**Affiliations:** Department of Experimental Biology, Faculty of Experimental Sciences, University of Jaén, Paraje de las Lagunillas, s/n, 23071, Jaén, Spain

**Keywords:** Rpb5, prefoldin-like, Bud27/URI, RNA polymerases, transcription

## Abstract

Rpb5 is one of the five common subunits to all eukaryotic RNA polymerases, which is conserved in archaea, but not in bacteria. Among these common subunits, it is the only one that is not interchangeable between yeasts and humans, and accounts for the functional incompatibility of yeast and human subunits. Rpb5 has been proposed to contribute to the gene-specific activation of RNA pol II, notably during the infectious cycle of the hepatitis B virus, and also to participate in general transcription mediated by all eukaryotic RNA pol. The structural analysis of Rpb5 and its interaction with different transcription factors, regulators and DNA, accounts for Rpb5 being necessary to maintain the correct conformation of the shelf module of RNA pol II, which favors the proper organization of the transcription bubble and the clamp closure of the enzyme.

In this work we provide details about subunit Rpb5's structure, conservation and the role it plays in transcription regulation by analyzing the different interactions with several factors, as well as its participation in the assembly of the three RNA pols, in cooperation with prefoldin-like Bud27/URI.

## 1. Rpb5 is a subunit of RNA polymerases

RNA polymerases (RNA pol) are the enzymes responsible for the specific synthesis of different types of RNAs. While the genome in bacteria and archaea is entirely transcribed by one single RNA pol, most eukaryotic cells use three distinct enzymes (RNA pol I, RNA pol II and RNA pol III), which carry out type-specific transcription programs in conjunction with different accessory factors. RNA pol I transcribes the precursor of the three largest rRNAs (35S rRNA), RNA pol II produces all mRNAs and many non coding RNAs, while RNA pol III synthesizes a set of small non translated RNAs, including all tRNAs, 5S rRNA and other short RNAs [1]–[7]. Moreover, two additional polymerases have been described in plants: RNA pol IV and RNA pol V. These enzymes synthesize small interfering RNAs (siRNAs) together with the long non coding RNAs involved in the development and response to environmental changes [8]–[10].

Eukaryotic RNA pols are characterized by their complex heteromultimeric composition. In yeast and other eukaryotes, RNA pol I, II, III are composed of 14, 12, and 17 subunits, respectively [4],[6],[11]. These heteromultimeric complexes share a conserved core of 12 subunits, five of which are common to the three enzymes (Rpb5, Rpb6, Rpb8, Rpb10, Rpb12; see Table 1) [12]–[15]. Notably, plant RNA pol IV and V contain different orthologs of some of these subunits [8]–[10], and trypanosomatid RNA pol has two isoforms of Rpb5 and Rpb6, one which represents the canonical RNA pol subunit [16] (Table 1).

**Table 1.** RNA pol common subunit composition.

		Eukaryotes			
Bacteria	Archaea	RNA pol I, II, III	RNA pol IV (plants)	RNA pol V (plants)	RNA pol trypanosome
-	Rpo5 (RpoH)	RPB5	[3]	NRPES5	RPB5/RPB5z
ω	Rpo6 (RpoK)	RPB6	[1]		RPB6/RPB6z
-	RpoG*	RPB8	[1]	[1]	
-	Rpo10 (RpoN)	RPB10			
-	Rp012 (RpoP)	RPB12			

For plants and trypanosome, subunits are indicated only when they change; the numbers in square brackets indicate the number of orthologues of RNA pol IV and RNA pol V subunits in plants with respect to these of RNA pol I, II and III. *Subunits RpoG have been identified only in some archaeal species [12]. Different names for common subunits of yeast RNA pol: Rpb5: ABC27; Rpb6: ABC23 or Rpo26; Rpb8: ABC14.5; Rpb10: ABC10β; Rpb12: ABC10α.

One of these RNA pol common subunits, Rpb5, is an evolutionarily highly conserved RNA pol component that is described as a key structural and functional component of all eukaryotic RNA pols, which suggests a more general role in transcription [13].

In this report we provide details about subunit Rpb5 and its relationship with prefoldin-like Bud27 and their roles in transcription regulation.

## 2. Rpb5 organization

**Figure 1.**
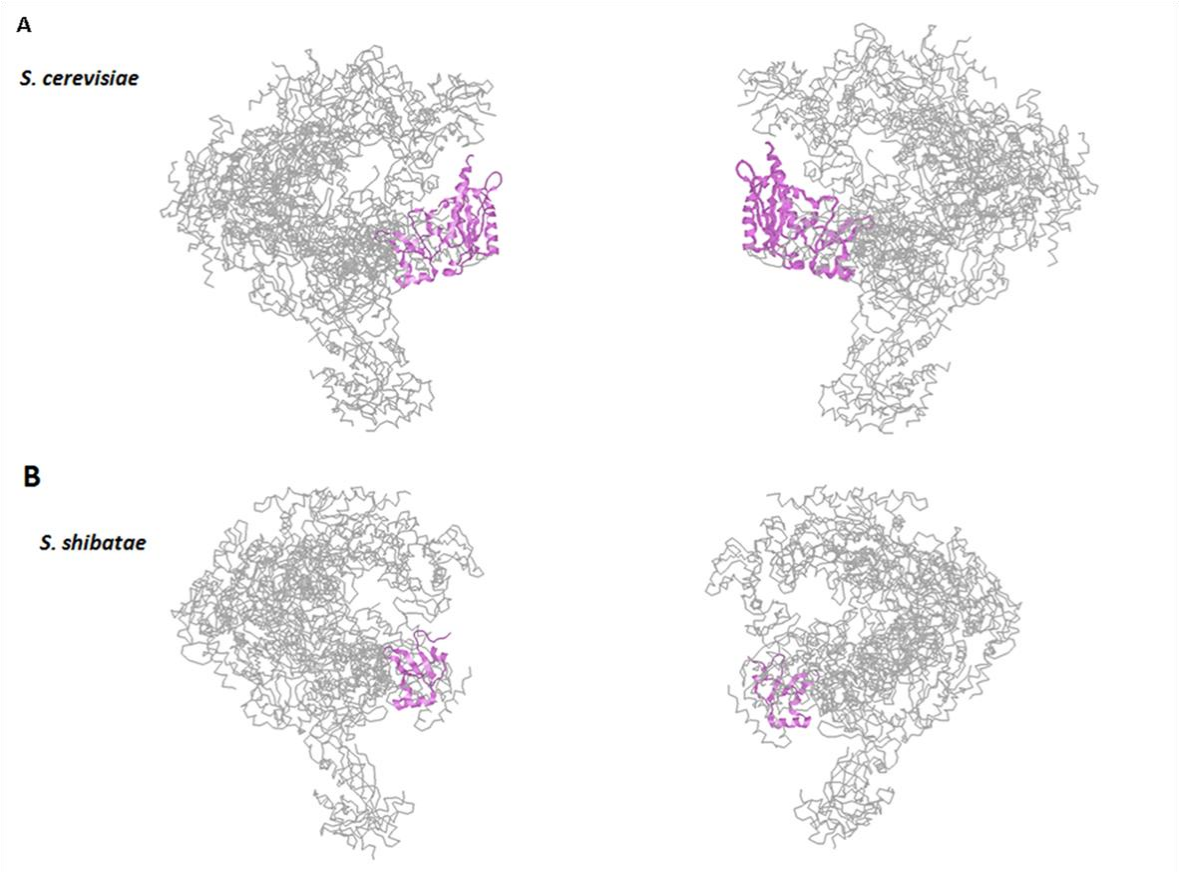
Structural view of Rpb5 and RpoH. A) Spatial organization of Rpb5 (violet) on the RNA pol II structure from *Saccharomyces cerevisiae* (pdb: wcm). B) Spatial organization of RpoH (violet) on the RNA pol structure from *Sulfolobus shibatae* (pdb: 2waq). Figures of the molecules were prepared with the RASMOL program.

**Figure 2.**
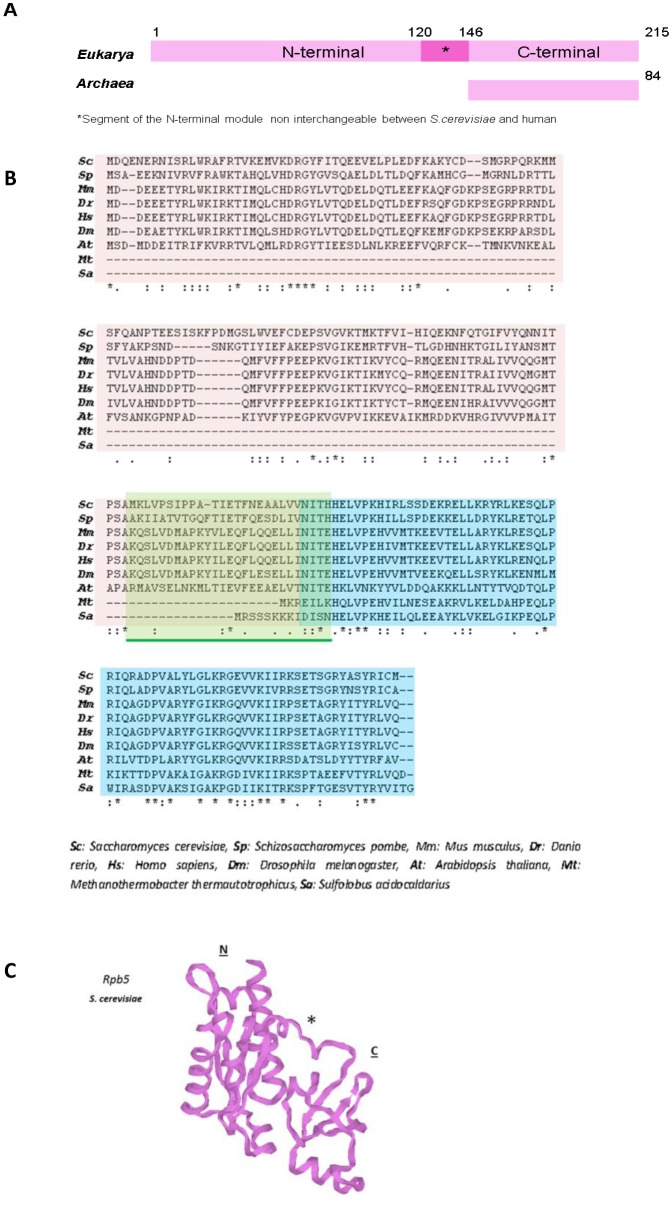
Comparison of Rpb5 and RpoH. (A) Schematic representation of eukaryotic Rpb5 and archaeal RpoH. The numbers on Rpb5 correspond to the amino acid positions in *Saccharomyces cerevisiae* and the numbers on RpoH to *Sulfolobus acidocaldarius*. (B) Comparison of Rpb5 and RpoH from different origins with clustal omega; *amino acid identity; “:” and “.” correspond to highly or less conserved amino acids, respectively. In clear brown, blue and green, the N-terminal, the C-terminal and the incompatibility domains, repectively. (C) Spatial organization of Rpb5 from *S. cerevisiae* (pdb: 1dzf). N: eukaryotic N-terminal domain (positions 1–142 in *S. cerevisiae*). C: C-terminal globe (positions 146–215 in *S. cerevisiae*). *Marks the segment of the N-terminal domain that is non interchangeable between *S. cerevisiae* and human.

In *S. cerevisiae*, Rpb5, also called ABC27, is located in the periphery on all three RNA pols and occupies the “lower” far-end of the DNA cleft [17]–[21] (Figure 1A). In budding yeast, Rpb5 consists of 215 amino acid residues with a molecular mass of 27 kDa [13],[14],[22],[23]. The strong evolutionary conservation of Rpb5 is evidenced by its homology with archaeal subunit H, also known as RpoH [1],[24]–[26], and to viral Rpb5-like subunits in nuclear and cytoplasmic DNA viruses [27],[28]. Notably, archaeal RpoH lacks the N-terminal domain present in the eukaryotic subunit and is also peripherally located on RNA pol (Figure 1B, Figure 2A and 2B). It is noteworthy that, unlike other RNA pol common subunits, the human Rpb5 homolog (with 44% identity and 80% similarity to the yeast subunit) is unable to complement the *RPB5* null allele in *S. cerevisiae* [13],[15].

Rpb5 shows a bipartite organization (Figure 2C) by combining two globular modules divided by a short hinge: an eukaryotic N-terminal domain (*jaw* domain), which corresponds to positions 1–142 in *S. cerevisiae*, and a C-terminal globe (*assembly* domain), which is largely conserved in all non bacterial enzymes [19],[24],[25],[29]–[31]. Both domains are essential *in vivo*, and are functionally exchangeable with their human homologs, except for a small central segment located between positions 121–146 in *S. cerevisiae*, which marks the end of the eukaryotic domain and extends to the first four amino acids of the C-terminal domain [13]. It is tempting to speculate that this small central region is necessary for N-terminal domain mobility. The N-terminal domain is moderately conserved (Figure 2B), but contains highly conserved sequence blocks and an important helix Rpb5-α1 that occupies the “*lower*” far end of the DNA *cleft*. Two prolines (P86 and P118 in *S. cerevisiae*) have been proposed to contact DNA. Moreover, the C-terminal module is highly conserved (positions 143–215 in *S. cerevisiae*) and binds a conserved fold on the largest subunits of RNA pols I, II and III (Rpa190, Rpb1 and Rpc160, respectively), which correspond to a bundle of four helices (Rpb1-α44/47) [13],[32]. Notably the C-terminal part of Rpb5 and archaeal RpoH are extremely similar in the tertiary structure, as shown by the superposition of both *S. cerevisiae* Rpb5 and *Sulfolobus solfataricus* RpoH archaeal subunits [33].

## 3. Rpb5 contacts DNA

The N-terminal domain of Rpb5 accounts for the Rpb5/DNA contacts ahead of the transcription fork (15–20 nucleotides) in *S. cerevisiae* RNA pol II [34],[35] and III [36]. This contact involves Pro118 in the amino-terminal region, which is positioned in the minor groove of the DNA double helix, Thr117 and Ser119. However, the previous mutant that contains the replacement of Pro118 with Thr, P118T, does not show any detectable growth defect, which suggests that Pro118 plays no major role in this contact [13]. In human RNA pol II, the DNA downstream of the transcription bubble contacts the *jaw* domain of Rpb5 through a conserved “TPSA domain”, apparently an interaction that has already been established during transcription initiation, and is maintained during the transition from initiation to elongation [11],[37]. Data are consistent with a model where the downstream DNA moves into the RNA pol II *cleft* by translocation along Rpb5 upon TFIIH action and DNA opening [37],[38]. In archaea, the Rpb5 homolog RpoH is also proximal to the downstream DNA in the preinitiation complex, and is important for both open complex formation and initial transcription [25],[33].

The peripheral localization of Rpb5 [13],[19]–[21] could enable interactions with chromatin or chromatin-associated proteins. Notably in *S. cerevisiae*, Rpb5 interacts with Rsc4, a subunit of the RSC complex (chromatin remodeler complex) that regulates transcription negatively or positively through its ability to mobilize nucleosomes [39]. Rpb5 could directly contact chromatin in elongating RNA polymerases through this interaction. It is tempting to speculate that this interaction is not maintained in archaea due to the lack of the N-terminal Rpb5 domain in the RpoH subunit, or this interaction could be established via a different mechanism since archaeal chromatin differs from that of eukaryote [40].

## 4. Rpb5 in transcriptional regulation

Many studies point out a role of Rpb5 in transcription regulation, but the specific role of Rpb5 in transcription remains unclear.

Its peripheral localization also enables interactions with general transcription factors or specific gene regulators. Accordingly a role for yeast Rpb5 in transcription activation has been previously proposed. The *in vitro* and *in vivo* analyses done with an Rpb5 and RNA pol II CTD mutants showing that Rpb5 and CTD had overlapping functions in the activation, account for this function [41].

As shown in Table 2, human Rpb5 has been shown to contact the transcriptional activator HBx (Hepatitis B virus X protein) and to participate in hepatitis B virus (HBV) infection [42],[43]. The interaction between HBx and the central part of Rpb5 modulate and stimulate transcription [42], but also requires the general transcription factor TFIIB, which establishes a trimeric interaction needed for HBx transactivation [43]. Rpb5 specifically binds the Unconventional Prefoldin Rpb5 Interactor (URI/RMP) *in vitro* and *in vivo* by negatively modulating transcription, and by probably interfering with the trimeric interaction among Rpb5, TFIIB and HBx [44]. Rpb5 directly binds RAP30, a subunit of the general transcription factor TFIIF, and this interaction is important for the association between RNA pol II and TFIIF [45],[46]. Notably, RAP30 and HBx bind Rpb5 in the same region, which suggests that these two factors compete for Rpb5 binding [45]. Furthermore, it has been proposed that TFIIF may cooperate with Rpb5 and TFIIB for the corepressor function of URI/RMP [47].

Human Rpb5 also interacts with the Human TATA-binding protein (TBP)-associated factor 68 (TAFII68), an RNA/ssDNA binding protein that was originally identified for its homology to the proto-oncogenes EWS (Ewing's Sarcoma) and TLS (Translocated in Liposarcoma; another member of the EWS gene family) [48]–[50]. Finally, Rpb5 physically interacts *in vitro* with TIP120 (TATA-binding protein-interacting protein 120) which stimulates the enzymatic activity of both RNA Pol I and II [51].

**Table 2.** Rpb5 interactors.

Interactor	Description	Organism	Reference
Rsc4	RSC chromatin remodeler	*S. cerevisiae*	[39]
HBx	Hepatitis B virus X protein	Human	[42]
TFIIB	General Transcription factor B	Human	[42]
RMP/URI/Bud27	Prefoldin-like	Human and yeast	[44],[55]
RPAP30	Subunit of general transcription factor TFIIF	Human	[45],[46]
TAFII68	Human TATA-binding protein (TBP)-associated factor 68 (TAFII68)	Human	[50]
TIP120	TATA-binding protein-interacting protein 120	Human	[51]

In yeast a major role has been described for Rpb5 in maintaining the proper organization of the RNA pol II structure around the transcription bubble [13], which falls in line with data that demonstrate the association of Rpb5 and RpoH with the DNA downstream of the transcription bubble [1],[34],[37],[52]. These data pointing to this interaction as a prerequisite in transcription initiation agrees with our recent work that demonstrated a specific role of Rpb5 in the transition from transcription initiation to elongation mediated by RNA pol II, by modulating the association with the elongation factor Spt4/5 and the backtracking activity. Based on our results, we propose that Rpb5 is necessary to maintain the correct conformation of the shelf module of the RNA pol II to favor the proper organization of the transcription bubble and the clamp closure. This implies the movement of Rpb4/7 and Spt5 association to thus allow the transition from transcription initiation to elongation [53].

In line with a role for Rpb5 that favors transcription elongation, loss of the Rpb5-Rsc4 interaction alters the chromatin structure in the promoter region of several RSC-regulated genes, and consequently impairs transcription [39].

## 5. Bud27/URI, a prefoldin-like component

Bud27 and its ortholog URI (Unconventional prefoldin RPB5-interactor), are members of the prefoldin (PFD) family of ATP-independent molecular chaperones in higher eukaryotes [54]. Bud27/URI was initially described as a protein that binds the Rpb5 subunit to all three nuclear RNA polymerases, and is considered to function as a scaffold protein capable of assembling additional members of the prefoldin (PDF) family in both human and yeast [44],[55].

In mammals, URI forms a complex with PFDN2, PFDN6, UXT, WDR92/Monad, PDRG1 and RPB5 [56], which is assumed to adopt a prefoldin-like structure and to cooperate with the cochaperone R2TP complex in the cytoplasmic assembly of RNA pol II [57]–[61]. Bud27 has also been found to be an interactor of Pfd2 and Pfd6 in *S. cerevisiae* [55]. Bud27 and URI have been proposed to be TOR (for Target of Rapamycin) pathway members in both *S. cerevisiae* and humans by participating in the gene expression controlled by TOR kinase [56]. Consequently, Bud27/URI could form part of a signaling pathway that regulates nutrient availability and gene expression.

Other functions for URI have been described. In human HEK-293 cell extracts, URI interacts with the human homolog of *S. cerevisiae* Paf1, a complex involved in cell-cycle control, RNA pol II phosphorylation, and histone modification during transcription elongation [54],[62]. The role of URI in the nucleus is not restricted to transcription. In fact it functions in DNA integrity maintenance in *C. elegans* [63], DNA damage in *Drosophila* [64], and also in tumorogenesis through DNA damage inhibition of *de novo* NAD + synthesis in mouse [65]. Furthermore, URI participates in oncogenic processes in human and mouse [65],[66].

Finally, *S. cerevisiae* Bud27 has been described to coordinate translation initiation and cotranslational quality control [67].

## 6. Bud27/URI cooperates with Rpb5

The importance of the Rpb5-Bud27 (RPB5-URI) interaction has been described in transcription regulation. In fact it was originally described how these two human proteins, in association with transcriptional activator HBx (Hepatitis B virus X protein), modulate its action [68] to favor hepatitis B viral infection [42],[44]. As a corepressor, URI competes with HBx to bind general transcription factor TFIIB, which cooperates with Rpb5. URI also regulates transcription through its association with general transcription factor TFIIF. Alternatively, TFIIF may cooperate with Rpb5 and TFIIB for URI to function as a corepressor [47].

The role of URI controlling androgen receptor (AR) transcription supports the idea of an RPB5-URI interaction, where Rpb5 could participate in regulating the androgen receptor in human cells [69].

Bud27 in *S. cerevisiae* has recently been described as the first protein to mediate the cytoplasmic assembly of the three RNA polymerases prior to their entry to the nucleus, a function that depends on Rpb5 [55]. A role for Bud27 in RNA pol III assembly has also been demonstrated [70], as well as the interaction between Rpb5 and assembly/import factor Rbs1 [71]. Consistently with this idea, a role for URI in human cells has been demonstrated in the assembly of the RNA pol II, found in association with the subassembly complexes that contain Rpb5 and R2TP [58],[59].

Notably, Bud27 participates in transcription elongation in *S. cerevisiae*, probably through the correct association with Sth1 (a RSC complex's subunit). We speculate that this action may involve a tripartite association among Bud27, Rpb5 and Sth1 [70],[72]. Furthermore, a functional interaction has been previously reported between Rpb5 and RSC [39].

## 7. Conclusion

Rpb5, as the only one of the five RNA polymerase common subunits to contact DNA and chromatin remodelers, may play a more relevant role than a general element in transcription. In fact its recently described participation in the transition from transcription initiation to elongation by modulating Spt5 elongation factor association and backtracking demonstrates a specific role of this subunit in RNA pol II activity. The association between Rpb5 and Bud27, a prefoldin-like protein that participates in RNA pols assembly, transcription, translation and the TOR pathway, among other important cellular processes, prompted us to speculate that this connection could regulates transcription, ribosomal biogenesis and growth. Notably URI, the human ortholog of Bud27, is altered in cancer processes.

The importance of these two proteins and their association in regulating important cellular processes is currently being studied.
